# Case Report: Cotard's Syndrome in Anti-N-methyl D-aspartate (NMDA) Receptor (Anti-NMDAR) Encephalitis

**DOI:** 10.3389/fpsyt.2022.779520

**Published:** 2022-05-04

**Authors:** Nur Iwana Abdul Taib, Suzaily Wahab, Ching Soong Khoo, Hui Jan Tan, Lydia Kamaruzaman, Luke Sy-Cherng Woon, Lydia Lay Yen Gan

**Affiliations:** ^1^Department of Psychological Medicine, Faculty of Medicine and Health Sciences, University Malaysia Sarawak, Kota Samarahan, Malaysia; ^2^Department of Psychiatry, Faculty of Medicine, Universiti Kebangsaan Malaysia, Kuala Lumpur, Malaysia; ^3^Neurology Unit, Department of Medicine, Universiti Kebangsaan Malaysia Medical Centre, Kuala Lumpur, Malaysia; ^4^Department of Medicine, Faculty of Medicine, University Kebangsaan Malaysia, Kuala Lumpur, Malaysia; ^5^Department of Pharmacy, Hospital Canselor Tuanku Muhriz, Kuala Lumpur, Malaysia

**Keywords:** case report, Cotard's syndrome, anti-NMDAR, nihilistic delusion, encephalitis, psychosis, neuropsychiatry

## Abstract

Cotard's syndrome is uncommon psychopathology among patients with psychotic illnesses. Limited cases had been reported regarding the occurrence of this syndrome in anti-NMDAR encephalitis which itself is a relatively new disease that often presents with florid psychotic symptoms. This poses difficulties in differentiating it from a primary psychiatric illness. Late recognition of anti-NMDAR encephalitis can lead to death as it can progress to autonomic instability in its natural course of illness. We report a patient who first presented with psychotic symptoms with initial negative findings from baseline investigations. Further investigation revealed anti NMDAR antibodies in the cerebrospinal fluid. Prompt treatment was initiated and despite early poor response to the first-line treatment with the development of allergic reaction, our patient recovered completely after 1 month of hospitalization. This case report aims to highlight the importance of early detection of anti-NMDAR encephalitis and the possibility of uncommon psychopathology such as Cotard's syndrome occurring in this disease.

## Introduction

Cotard's syndrome is a rare neuropsychiatric condition characterized by nihilistic delusion. Patients typically believe that they are dead or have lost their organs and body parts (1). This syndrome can exist in patients with depression, schizophrenia, or psychotic disorder caused by a general medical condition. Anti-N-methyl D-aspartate (NMDA) receptor (anti-NMDAR) encephalitis is an autoimmune disease arising from the generation of IgG antibodies targeting the NRI subunit of the NMDA in the brain. It was reported that about 50% of encephalitis admissions over a year in a tertiary center in Malaysia were caused by it (2). There is a constellation of symptoms that can manifest in anti-NMDAR encephalitis, including autonomic instability, psychiatric, and neurological symptoms. These varied manifestations pose a unique challenge for clinicians to diagnose this disease during the initial encounter at the emergency department or outpatient setting. Due to the early presentation of neuropsychiatric symptoms in the illness, ~77% of patients are first assessed by a psychiatrist (3). Other than a positive cerebrospinal fluid (CSF) finding of anti NMDAR antibodies, there are no other specific investigations to aid in the diagnosis. It is thus crucial to have a high index of suspicion of this disease when managing patients with an acute onset of psychiatric symptoms. Co-occurrences of Cotard's syndrome and anti-NMDAR encephalitis are uncommon as highlighted by another case report of a young man with similar presentation and the importance of early detection (4). We are presenting a case of a young lady with anti-NMDAR encephalitis manifesting Cotard's syndrome who eventually achieved complete recovery from her illness despite initial poor response to first-line treatments.

## Case report

A 24-year-old woman of Chinese descent without underlying medical or psychiatric illnesses was brought to the emergency department for a 5-day history of acute behavioral change. Prior to the onset of symptoms, there were no known stressors or significant life events and she was reported to be completed well with no fever, constitutional symptoms or infective symptoms. When the illness started, she became socially withdrawn with periods of restlessness and agitation at home, insomnia, and irrelevant speech. She presented with the classic symptoms of Cotard's syndrome, namely nihilistic delusions, besides delusion of control and auditory hallucination. Nihilistic belief related to the body was observed as she reported that “my body is not real, filled with grave dirt as I am already dead”. There was a nihilistic delusion regarding her existence as she reported that “I am already dead and buried in the grave”, and “I am in the afterlife, everyone here is already dead”. She believed she was a walking corpse as she roamed around in the hospital ward. The beliefs were strongly held despite attempts to challenge them. She behaved in a disorganized manner in response to her psychosis, spitting out saliva inappropriately in the belief that she was removing grave dirt and semen from her body. There was echolalia at times. Orientation to time, place, and person fluctuated. Recent memory was affected. For instance, she could not recall who had brought her to the hospital and whether she had her magnetic resonance imaging (MRI) done. Her affect was inappropriate and incongruent with her thoughts. Physical examination revealed no significant abnormalities and vital signs including blood pressure, pulse rate, and temperature were normal in the initial assessment. Full blood count showed a raised total white cell count but infectious markers such as erythrocyte sedimentation rate (ESR) and C-reactive protein (CRP) were not raised. Computerized tomography (CT) of the brain was normal. The patient was admitted to the psychiatric ward for further management. Oral tablet olanzapine 10 mg nocte was commenced to treat her psychotic symptoms.

About 1 week after her initial presentation, the patient developed a complex seizure with tonic movements over bilateral upper and lower limbs and up-rolling of eyeballs that lasted for 1 min. The patient started having low-grade fever, sweating, and tachycardia. Olanzapine was withdrawn at this moment because of the less prominent psychotic symptoms and the emerging neurological symptoms raising the suspicion of the neuroleptic malignant syndrome. The patient was referred to the neurology team in the hospital for co-management. A contrasted MRI brain revealed no significant abnormalities, and an electroencephalogram (EEG) was normal. Despite the lack of positive findings from the imaging study, a lumbar puncture was done, which yielded a positive anti NMDAR antibody titer 1:1 with a negative infective screening. An assessment done by the gynecology team ruled out ovarian teratoma as the etiology. A 5-day course of intravenous methylprednisolone was administered but produced an inadequate clinical response. Thus, intravenous immunoglobulin (IVIG) treatment was initiated.

The patient was planned for IVIG infusion at the dosage of 0.4 g/kg once daily for 5 days along with intravenous methylprednisolone 500 mg once daily. However, during the first session of IVIG infusion, she developed fever, tachycardia, dizziness, headache, chills, chest pain, nausea, vomiting, and diarrhea. Maculopapular rashes also appeared around her neck. The infusion was immediately suspended for suspected infusion-related allergic reaction, and she was transferred to the high dependency unit for close observation. The infusion-related symptoms gradually subsided.

During the treatment with IVIG, the patient progressed into a catatonic state with resistance to passive movements, not responding to the command, and not eye-opening with fluctuating consciousness. Physical examination revealed hypertonicity of all four limbs and neck muscle with oro-buccal dyskinesia involving clenching of teeth and repetitive chewing movements. There was autonomic instability with periods of hypertension, tachycardia, and fever. A repeated EEG showed mild diffuse generalized cerebral disturbances with excessive Theta activity that supported occurrence mild encephalopathy in this patient.

As the patient developed allergic reactions to IVIG, she was treated with therapeutic plasma exchange (TPE) instead. TPE was done every other day, with a total of five sessions conducted. She was initially planned to receive at least five sessions of plasma exchange, depending on her clinical condition. At the fourth plasma exchange, the patient started to show improvement as she was able to obey simple commands. TPE was terminated after the fifth session when the patient developed right common iliac vein thrombosis, requiring the removal of her femoral vein catheter. By now at almost 4 weeks of illness, the patient was able to cooperate with the treating team. She could hold conversations properly. There was a complete resolution of psychotic symptoms. She was able to perform activities of daily living (ADL) independently without neurological deficits. After a month of hospitalization, the patient was discharged home with oral azathioprine and prednisolone, which were started 3 days before her discharge. Antipsychotic was not reinitiated as the psychotic symptoms had fully remitted.

The patient was followed up in the next 4 months. By 2 months after her discharge, the patient remained unable to recall the events that occurred during her hospital admission, but the patient and her family reported complete recovery from her symptoms. The delusions had resolved fully. She was able to return to her baseline level of functioning, with plans to resume working soon. The relevant events and treatments were summarized in Table 1.

**Table 1:** Timeline of events and treatments.

	**Events**	**Treatment**
Day 1	Onset of behavioral change with acute psychosis	
Day 5	Presented to Emergency Department	Initiated with Oral Olanzapine 10 mg nocte
Day 7	Developed complex seizure, low grade fever, sweating and tachycardia	Oral olanzapine were withdrawn
Day 8	No abnormalities on MRI brain and EEG Lumbar puncture yielded positive anti NMDAR antibody titer 1:1	IV methylprednisolone started for 5 days
Day 12	Inadequate clinical response to IV methyprednisolone	Intravenous immunoglobulin (IVIG) treatment was initiated
	During the first session of IVIG infusion, she developed suspected infusion-related allergic reaction Progressed into a catatonic state with autonomic instability Repeated EEG showed mild diffuse generalized cerebral disturbances with excessive Theta activity	Treated with therapeutic plasma exchange (TPE) every other day, with a total of five sessions conducted
Day 23	Developed right common iliac vein thrombosis, requiring the removal of her femoral vein catheter TPE was terminated after the fifth session	
Day 25	Condition improved	
1 month	Discharged home	Oral azathioprine and prednisolone
2 months	Outpatient review: Unable to recall the events that occurred during her hospital admission, but the patient and her family reported complete recovery from her symptoms. The delusions had resolved fully. She was able to return to her baseline level of functioning, with plans to resume working soon	

## Discussion

Cotard's syndrome was initially described in 1880. It manifests as nihilistic delusions which vary from denial of the existence of body parts to negation of self-existence (5). Nevertheless, very little is understood about this disorder. Although it is most commonly associated with psychiatric disease, several reports have recounted its occurrence in anti-NMDAR encephalitis (5, 6). A recent study by Ramirez-Bermudez and colleagues provides some insights into Cotard's syndrome in anti-NMDAR encephalitis from the imaging studies of two similar cases that showed a bilateral hemispheric pattern of hypometabolism in posterior regions of the brain (5). In our patient, Cotard's syndrome was very prominent as it appeared to be the main theme of her psychosis as she had the delusion that she was already dead and everyone around her was also dead. She reacted to the delusion by spitting out saliva which she believed to be grave dirt from her body. Whereas, earlier published case had reported an atypical presentation of anti-NMDAR encephalitis which include features of Cotard's syndrome together with Capgras delusion, Capgras delusion was comparatively absent in our patient (7).

Anti-NMDAR encephalitis is a relatively new disease after it was first discovered in 2007, but it has since significantly affected our approach to first-episode psychosis (8). Severe behavioral changes due to this illness are almost similar to a schizophrenia-like illness, suggesting that this disease might define a small group of patients being misdiagnosed as having a primary psychiatric disease (9, 10). Females are four times more at risk to be affected, and the most common age group is between 25 and 35 years old (11). Although it is a medical condition, the majority of the patients are first seen by psychiatrists due to the early presentation of severe neuropsychiatric symptoms in the illness (3). In our patient's case, a high index of suspicion for anti-NMDAR encephalitis is due to her profile as being a young female with severe first-episode psychosis. The recognition of such a clinical profile by the treating team allowed early treatment to be initiated.

Anti-NMDAR encephalitis in most patients starts with a prodromal phase which resembles a common viral infection lasting 5 to 14 days (12, 13). The psychiatric symptoms of anti-NMDAR encephalitis patients are diverse, involving emotional and behavioral disturbances such as apathy, fear, hypersexuality, wandering, amnesia, severe insomnia, depression, decreased cognitive skills, and psychosis (11, 14). Compared to schizophrenia, which has more prevalent positive symptoms, patients with anti-NMDAR encephalitis display both positive and negative symptoms (11). These non-specific psychiatric symptoms can appear rapidly within days or weeks in patients with no prior psychiatric diagnosis (11). This is a major differentiating point as the onset of the psychotic phase in anti-NMDAR encephalitis occurs quickly in contrast to the slow progression of primary psychiatric disease. Fluctuations in mental status predominate throughout the course of illness, thus the psychiatric presentation is best seen as delirium or acute confusional state that develops over a short time, which vacillates over the course of the day and manifests as changes in cognition, affect, behavior, and perception. Many patients are admitted to the psychiatric ward due to severe symptomatology and spend weeks for symptomatic management. We can observe severe psychotic symptoms in our current case presentation. The patient had the delusion of control which resembled schizophrenia as well as nihilistic delusion which was the Cotard's syndrome. The severity of these symptoms might an inexperienced clinician into making a diagnosis of a primary psychiatric disorder. However, the acute onset of the symptoms within the spans of a few days of such intensity would warrant further investigations for an organic cause.

The unresponsive phase of anti-NMDAR encephalitis follows the psychotic phase and is characterized by decreased motor activity, mutism, and catatonia (11). Patients may also demonstrate stereotyped athetotic movements (14). In the hyperkinetic phase, there are prominent movement disorders and autonomic instability. The common movement disorder in this phase is oro-lingual dyskinesias but a diverse range of movement disorders can also be present (11, 15). With adequate immunotherapy and supportive care, a patient can progress to the recovery phase.

Classic progression of the anti-NMDAR encephalitis was seen in our patient's presentation. Information on her prodromal phase was limited as the main historian during her acute illness was her family given her inability to provide information when she was in her psychotic state. Nonetheless, her disease progression was closely monitored by the treating team, especially when she entered the unresponsive and hyperkinetic phases. The treating team foresaw the progression into these phases; hence the patient was nursed in a high dependency unit to provide adequate support should any complications arise.

Confirmation of the diagnosis of anti-NMDAR encephalitis is mainly from the detection of the NR1 IgG antibodies from serum or CSF (16). Other findings in CSF include oligoclonal bands, normal or mildly increased protein concentration, and moderate lymphocytic pleocytosis (17, 18). About 90% of patients with anti-NMDAR encephalitis had abnormal EEG but it is not diagnostic. The EEG characteristic was described as “extreme delta brush” and it is associated with prolonged hospitalization (19). However, a normal EEG finding is not an exclusion for this diagnosis. Brain MRI can be normal in up to half of the affected patients or it may show transitory contrast-enhancing lesions in the cortical or subcortical regions (20, 21). Despite lacking in diagnostic value, it would be useful to exclude space-occupying lesions and to screen for other organic causes. In addition, female patients should be screened for ovarian teratoma due to its high association with anti-NMDAR encephalitis after the diagnosis is confirmed. An example of negative imaging and EEG findings can be seen in our case, as only the CSF sample yielded a positive anti-NMDAR titer. This could have been a missed diagnosis if the CSF sample were not sent for anti-NMDAR titer testing. An inexperienced clinician may rule out organicity as a cause after getting negative imaging and EEG results. There are red flags clinicians can look for in arousing suspicion for this diagnosis. The signs are rapid symptom progression over days to weeks, absence of prior psychiatric history, presence of benign teratoma or other tumors, or a viral infection, the coexistence of very mild neurologic symptoms such as facial twitching, symptoms refractory to antipsychotics agents, abnormal brain MRI or EEG findings, and cerebrospinal fluid pleocytosis (22).

The mainstay of medical therapy is potent immunosuppressants including high-dose intravenous corticosteroids, intravenous immunoglobulin, anti-inflammatory agents, plasma exchange, and monoclonal antibodies directed against CD-20 B-lymphocytes (14). TPE or plasmapheresis is a treatment modality that involves passing a patient's plasma through a plasma filter to remove the undesirable antibodies. The amount of plasma removed is then replaced by either human albumin or fresh frozen plasma. The volume of plasma exchange varies from one to two times plasma volume. To the best of the authors' knowledge, there are no studies on the ideal amount of plasma volume and the total number of plasma exchange cycles required in TPE for anti-NMDAR encephalitis. At our center, we usually perform five or more plasma exchange (PEX) cycles, depending on the patient's clinical improvement. The common complications of PEX include catheter-related injuries such as bleeding, hematoma, infection, and venous thrombosis. Treatment-related complications are fever, coagulopathy, hemolysis, hypocalcemia, and allergic reactions to either albumin or blood products (23).

Most IVIG reactions are rate-related, mild, and occur in only 5–15% of infusions. They are typically characterized by back or abdominal pain, nausea, breathing difficulties, chills, flushing, rash, anxiety, low-grade fever, arthralgia, myalgias, and headache (24, 25). Adverse reactions are more likely to occur in patients who have not previously received IVIG. The Immune Deficiency Foundation survey found that 34% of reactions occurred during the first infusion of an IVIG product (26). Patients with underlying chronic inflammation and those with active or recent bacterial infection are also at higher risks. Patients with IgA deficiency may be predisposed to anaphylaxis as well (27). The reactions to IVIG may be due to complement activity caused by immune complexes that form between infused antibodies and antigens of infectious agents in the patient, thus manifesting as anaphylaxis/anaphylactic reactions upon exposure to IVIG therapy, which contains variable concentrations of contaminating IgA (28). Slowing or stopping the infusion for 15–30 min will reverse many reactions. Pre-treatment with NSAIDs, acetaminophen (15 mg/kg/dose), diphenhydramine (1 mg/kg/dose), or alternatively a non-sedating antihistamine and/or hydrocortisone (6 mg/kg/dose; maximum, 100 mg) 1 h before the infusion may prevent adverse reactions. Oral hydration before the infusion is often helpful. In our case, the patient was given proper pre-treatment before the IVIG infusion but still suffered from an anaphylactic reaction. Thus, it cannot be overemphasized that extra vigilance for adverse reactions is always required when administering IVIG to a patient for the first time. The patient scored 5 on the Naranjo scale and was given an allergy card.

There is limited literature on the use of psychiatric medications in the management of anti-NMDAR encephalitis. Nevertheless, general principles in managing psychiatric symptoms also apply. Both typical or atypical antipsychotic medications have been used either alone or in combinations to manage positive symptoms such as hallucination, delusion, and aggression in anti-NMDAR encephalitis (29). However, highly potent dopamine antagonists can cause extrapyramidal symptoms (EPS), which can worsen agitation and mimic the dyskinesia from the disease itself (29). Antipsychotics must be used with caution due to multiple reports of a heightened risk of antipsychotics intolerance such as neuroleptic malignant syndrome and EPS in patients with anti-NMDAR encephalitis (30–34). It has been suggested that signs of antipsychotic intolerance should raise suspicion for anti-NMDAR encephalitis (34). Currently there are no specific guidelines for the use of psychiatric medications in anti-NMDAR encephalitis, but clinical experience suggests the use of highly sedating medications such as quetiapine, chlorpromazine, valproic acid, and benzodiazepines, while high-potency antipsychotics like haloperidol have been observed to worsen motor symptoms in patients (35).

The prognosis of anti-NMDAR encephalitis is good with prompt and aggressive treatment (14). However, if treatment is delayed or ineffective, the mortality rate may be as high as 100% due to autonomic instability or complications of prolonged hospital admission and cardio-respiratory support (12). Hospital stay ranges from 2 to 14 months (36). The patient may not always return to their premorbid level of motor function and cognition, and it may take 3 years or longer to recover (14). About 20 to 25% of patients had relapses occurring at intervals between 3 months to 9 years after the initial presentation (14). Early initiation of immunosuppressants prompted by the high index of suspicion of anti-NMDAR encephalitis improved the prognostic outlook of our patient despite the treatment-associated complications.

This case report sheds some useful insights on the relatively rare co-occurrence of Cotard's syndrome and anti-NMDAR encephalitis. Even though experiencing treatment-related complications and receiving only a week of antipsychotic therapy, the patient managed to make a full recovery. She did not require long-term antipsychotic medication to remain well. The main good prognostic factor in this patient is the early suspicion of this condition. This was despite her non-specific clinical presentation and negative initial investigations. In conclusion, we hope to highlight the need for early suspicion of the diagnosis of anti-NMDAR encephalitis when encountering patients with acute onset of Cotard's syndrome, as early initiation of treatment will ensure a good outcome for the patient.

## Data Availability Statement

The original contributions presented in the study are included in the article/Supplementary Material, further inquiries can be directed to the corresponding author.

## Ethics Statement

Written informed consent was obtained from the individual(s) for the publication of any potentially identifiable images or data included in this article.

## Author Contributions

All authors listed have made a substantial, direct, and intellectual contribution to the work and approved it for publication.

## Conflict of Interest

The authors declare that the research was conducted in the absence of any commercial or financial relationships that could be construed as a potential conflict of interest.

## Publisher's Note

All claims expressed in this article are solely those of the authors and do not necessarily represent those of their affiliated organizations, or those of the publisher, the editors and the reviewers. Any product that may be evaluated in this article, or claim that may be made by its manufacturer, is not guaranteed or endorsed by the publisher.
