# Supplementary material for: Case Report: Cotard's Syndrome in Anti-N-methyl D-aspartate (NMDA) Receptor (Anti-NMDAR) Encephalitis
Source: Front Psychiatry. 2022 May 4;13:779520. doi: 10.3389/fpsyt.2022.779520 (PMC9114484; doi:10.3389/fpsyt.2022.779520)
Supplement: Supplementary file 1 [file Table_1.pdf]

SUPPLEMENTARY DOCUMENT

**INVESTIGATION**	**RESULTS**
Full blood count:<br>Hb<br>TWC<br>Platelet	 13.6<br>13.4<br>398
CRP	0.08
ESR	18
Creatine Kinase	200
Sr ANA	Positive, titre 1:80 Speckled
dsDNA	11.2 (<30)
C3/C4	122/27
Paraneoplastic Antibodies:<br>pANCA, cANCA, CA-125, CEA	 normal
CSF biochemistry:<br><br>Opening pressure<br>Protein<br>Glucose	 <br>10cmH2O, clear fluid<br>65 (Total protein 75)<br>4.6 (RBS 4.1)
CSF autoimmune profile:<br><br>Anti NMDAR<br>Anti AMPA 1/2<br>Anti GABA B<br>Anti DPPX<br>Anti LG11<br>Anti CA SPR2	 <br>Positive 1:1<br>Negative<br>Negative<br>Negative<br>Negative<br>Negative
CSF viral pathogen panel:<br><br>HSV1, HSV 2, HHV5, HHV7, Parvovirus B19, VZV, CMV, Mumps virus, Enterovirus, Adenovirus, Human Parechovirus RNA, EBV	 <br>Not detected
EEG x1	Normal

EEG x2 (one week later)	Mild diffuse generalized cerebral disturbances by excessive Theta activity.
MRI Brain contrast	No significant abnormalities
USG Abdomen	No evidence of adnexal mass.
CT TAP	Left ovarian cyst (follicular), no signs of malignancy.
